# Strangulation-Induced Thyrotoxicosis in a Patient with Undiagnosed Underlying Graves' Disease

**DOI:** 10.1155/2020/7986581

**Published:** 2020-03-19

**Authors:** Theresa Lanham, Abigayle Sullivan, Erik Lanham, Anthony Donato

**Affiliations:** ^1^Reading Hospital, Wyomissing, USA; ^2^Jefferson Health, Philadelphia, USA

## Abstract

Thyrotoxicosis is a constellation of symptoms including palpitations, tremors, agitation, and heat intolerance, caused by excess thyroid hormone. It can be life-threatening in its most serious form. We present a rare case of thyrotoxicosis provoked by mechanical trauma to the neck via strangulation in a young female with a history of self-resolving postpartum symptoms of hyperthyroidism one year prior, but no formal diagnosis of thyroid dysfunction. Although hyperthyroidism and posttraumatic stress have many similar features, thyroid storm is a life-threatening disorder that needs immediate intervention.

## 1. Introduction

Thyrotoxicosis is a constellation of symptoms including palpitations, tremors, agitation, and heat intolerance caused by excess thyroid hormone [1]. Untreated, it can lead to life-threatening thyroid storm which can be assessed clinically using the Burch–Wartofsky Point Scale (BWPS) [2]. The most common etiologies of hyperthyroidism include Graves' disease and toxic thyroid nodules, but it can also uncommonly be caused by excess iodine, germ cell tumors, surgery, infection, or mechanical trauma to the thyroid gland [1]. We present a case of thyrotoxicosis provoked by strangulation in the setting of previously undiagnosed, subclinical Graves' disease.

## 2. Case

A 33-year-old female smoker with a history of postpartum weight loss, tremors, and goiter one year prior presented with dyspnea, progressive neck pain, and swelling two days after being strangled by her boyfriend. She endorsed palpitations, chest tightness, tremors, anxiety, and poor sleep with nightmares since the assault. She also complained of difficulty swallowing. She denied diarrhea. She denied a history of radiation exposure or thyroid cancer. On exam, the patient was afebrile, normotensive (129/87 mmHg), and tachycardic (130 bpm). She had no altered mentation, but anxiety and a fine intention tremor were noted. Palpation of the thyroid gland demonstrated diffuse tender thyromegaly (2.5 times the normal size) with abrasion marks and bruises on her neck noted. She did not exhibit any pretibial or ophthalmic changes. Burch–Wartofsky score was 5. No prior thyroid function tests (TFTs) were on record. Labs were remarkable for a thyroid stimulating hormone (TSH) < 0.005 (ref: 0.45–5.330) uIU/mL, free T4: 5.55 (ref: 0.58–1.64) ng/dL, and free T3: 24.35 (ref: 2.20–4.10) pg/mL, and thyroid stimulating immunoglobulin: 144 (ref: ≤122)% and thyroglobulin 296 (ref: 1.6–50.0) ng/mL. Antithyroid peroxidase antibody: 38 (ref: ≤8) IU/mL. C-reactive protein was within normal limits. Thyroid ultrasound revealed a diffuse, heterogeneous thyroid swelling with hypervascularity. Computed tomography (CT) neck without contrast showed an enlarged thyroid without mass effect. The patient was started on methimazole 10 mg daily, propranolol 40 mg TID, and a prednisone 40 mg daily tapered over eight days. Two months after initial presentation, repeat TFTs demonstrated a TSH of 0.416 uIU/mL, free T4: 0.33 ng/dL, and free T3: 3.23 pg/mL. Nuclear medicine thyroid uptake and imaging revealed a diffusely enlarged thyroid gland without any nodules. The 5-hour and 24-hour uptake were 43.1% and 52.3%, respectively, consistent with Graves' disease. The patient continues to require methimazole 10 mg daily with normalization of TFTs (TSH: 2.066 uIU/mL, free: T4 0.62 ng/dL, and total: T3 1.1 (ref: 0.9–1.8) ng/dL) 6 months after hospitalization, and it is being planned for a radioiodine ablation procedure.

## 3. Discussion

Hyperthyroidism impacts the body negatively in many ways and can lead to compromise in hemodynamic stability and multiorgan failure [3]. Presentation may be quite variable clinically, but the most serious form is termed thyroid storm which has a mortality rate of 10–20% [4]. Typical presentation of thyroid storm includes fever, tachycardia, agitation or change in mental status, and gastrointestinal upset in a patient with evidence of hyperthyroidism biochemically [1, 2]. From a cardiac perspective alone, elevated thyroid hormone levels can increase induced tachyarrhythmias, valvulopathies, and cardiomyopathies. As such, patients may present in heart failure or atrial fibrillation [5]. The Burch–Wartofsky Point Scale (BWPS) can help differentiate hyperthyroidism or thyrotoxicosis from thyroid storm based on clinical symptoms, although it should only serve to help clinicians with high suspicion as it is not diagnostic by itself [2, 6].

Thyroid storm is rare, but is most commonly seen in patients with underlying Graves' disease [1]. Mechanical trauma precipitating thyrotoxicosis and thyroid storm has been described by numerous reports, but only two previous cases of strangulation-induced thyrotoxicosis have been published as per our literature review, one similar to our case with underlying Graves' disease [7] and one without [8]. In the patient described above, Graves' disease likely predisposed to thyrotoxicosis which was precipitated by blunt force trauma to the neck. The proposed pathophysiology states that mechanical stimulation of the thyroid gland causes excess release of preformed thyroid hormone in combination with increased catecholamine response as the result of the assault and psychologic stress that follows [7]. In fact, it is possible that psychological stress alone without the mechanical trauma could precipitate thyrotoxicosis in a patient with underlying Graves' disease. While disputed by some [9], there are studies that suggest stress and traumatic life events may be risk factors for and can even trigger Grave's disease [10–14].

In patients with mechanical trauma to the neck and tachycardia out of proportion to expected, physicians must consider thyrotoxicosis or thyroid storm. Although hyperthyroidism and posttraumatic stress have many similar features, thyroid storm needs immediate intervention to prevent mortality. Treatment includes methimazole to inhibit the synthesis of thyroid hormone, beta-blockade for symptomatic control, and steroids to reduce conversion of T4 to T3 peripherally [3, 6].
